# Should Sickle Cell Disease Be Considered a Cancer Predisposition Syndrome?

**DOI:** 10.3390/children13050683

**Published:** 2026-05-16

**Authors:** Elise Casadessus, Yves Pastore, Thomas Pincez

**Affiliations:** 1 Division of Pediatric Hematology-Oncology, CHU Sainte-Justine, Montreal, QC H3T 1C5, Canada; elise.casadessus@umontreal.ca (E.C.); yves.pastore@umontreal.ca (Y.P.); 2 Faculty of Medicine, Université de Montréal, Montreal, QC H3T 1J4, Canada; 3 CHU Sainte-Justine Azrieli Research Center, Montreal, QC H3T 1C5, Canada

**Keywords:** sickle cell disease, acute myeloid leukemia, cancer predisposition syndromes, clonal hematopoiesis, leukemogenesis

## Abstract

**Highlights:**

**What are the main findings?**

• Sickle cell disease and cancer predisposition syndrome share some similarities.
• Nevertheless, differences are substantial, with the main one being the lower relative risk of malignancy in individuals with sickle cell disease than in those with cancer predisposition syndrome.

**What are the implication of the main findings?**

• Absolute risk of malignancy is low in individuals with sickle cell disease, but clinicians should be aware of this rare event.
• The specificities of sickle cell disease regarding the risk of malignancy should be considered.

**Abstract:**

Among the many complications that can occur in individuals with sickle cell disease (SCD), several studies have suspected an increased risk of cancer. While the effect of SCD on solid tumors remains unclear, multiple studies support a higher incidence of leukemia, especially acute myeloid leukemia (AML). This risk seems to appear in childhood and persist throughout life. Based on these features, should SCD be considered a cancer predisposition syndrome? Here, we explore this question by comparing the characteristics of SCD-associated AML and cancer predisposition syndromes. We show that some features are similar. As in cancer predisposition syndrome, increased cancer risk in SCD appears to be restricted to a defined type of malignancy. SCD-associated AML also has molecular specificities reminiscent of therapy-related AML. Many of the mechanisms contributing to SCD-associated leukemogenesis have been reported in cancer predisposition syndromes, including ineffective erythropoiesis, increased cell renewal, chronic inflammation, and oxidative stress. Nevertheless, SCD presents a unique combination of factors, and their magnitude may greatly vary from one individual to another. Strikingly, the relative risk of cancer in SCD is much lower than most cancer predisposition syndromes and closer to those conferred by common variations. This is a major difference, and indeed, the absolute risk of malignancy in individuals with SCD appears to be low. Moreover, SCD has great clinical variability, and the factors influencing AML risk are unclear. In sum, SCD has many specificities compared to cancer predisposition syndromes that should be considered and investigated. Clinicians should be aware of the increased risk of AML in patients’ management and counseling.

## 1. Introduction

Sickle cell disease (SCD) is a hereditary hemoglobinopathy characterized by chronic hemolysis, vaso-occlusion, and multisystem complications. Beyond its well-established clinical manifestations, SCD is increasingly recognized as a condition associated with alterations in hematopoiesis and immune function [1,2,3].

Over the past decade, accumulating epidemiological and clinical evidence has suggested a potential association between SCD and an increased risk of malignancies, particularly hematological cancers such as acute myeloid leukemia (AML) [4,5]. Earlier reports first raised the possibility of an association between SCD and malignancy [6]. This observation was initially unexpected, as SCD has traditionally been conceptualized as a disorder primarily affecting mature red blood cells rather than hematopoietic stem and progenitor cells [3].

Recent advances in the understanding of SCD pathophysiology, including chronic inflammation, oxidative stress, ineffective erythropoiesis, and clonal hematopoiesis (CH), have provided a biological framework supporting a potential role for SCD in carcinogenesis [3,7,8]. In our previous work, we reviewed these mechanisms and highlighted the emerging oncogenic landscape associated with SCD [9].

However, whether SCD should be conceptualized as a cancer predisposition syndrome remains an open and insufficiently addressed question. Cancer predisposition syndromes are defined by a set of clinical and biological features, including early age at cancer onset, characteristic tumor spectra, specific underlying mechanisms, and a significantly increased lifetime risk of malignancy.

SCD encompasses a group of genotypes, including homozygous hemoglobin S (HbSS) and compound heterozygous forms such as HbSC and HbS/β-thalassemia, which may differ in clinical severity. In the present work, we primarily refer to the more severe HbSS genotype, as most available data on cancer risk have been generated in this population.

In the present study, we aim to address this question using a structured, criteria-based approach. By systematically comparing SCD with established cancer predisposition syndromes across epidemiological, clinical, and biological dimensions, we seek to determine to what extent SCD fulfills the defining features of a cancer predisposition syndrome, and to better position SCD within the broader landscape of cancer susceptibility.

## 2. Cancer Predisposition Syndromes: Conceptual Framework for Cancer Risk

Cancer predisposition syndromes are constitutional genetic conditions caused by germline pathogenic variants that increase an individual’s lifetime risk of malignancy [1,2,3,4]. Although there is no dedicated classification, approximately 100 cancer predisposition syndromes have been described [5,7]. Many of these conditions are autosomal dominant and caused by heterozygous (monoallelic) pathogenic variants (e.g., Neurofibromatosis type 1). A malignancy can develop when additional variants occur, frequently in the same gene as the one carrying the germline variant, but not exclusively. Other cancer predisposition syndromes are autosomal recessive diseases due to biallelic variants (e.g., Ataxia-Telangiectasia). Although identified for a long time in families with a strong history of malignancies, the contribution of cancer predisposition syndromes to sporadic malignancies is increasingly recognized. The recent expansion in the use of germline sequencing in pediatric oncology has revealed that approximately 8–10% of children with cancer harbor pathogenic variants in known cancer predisposition genes, highlighting the substantial contribution of inherited susceptibility to pediatric malignancies [1]. There are no universal criteria to define cancer predisposition syndromes, although some specific cancer predisposition syndromes have their own criteria, such as Li-Fraumeni syndrome. Most cancer predisposition syndromes share similar features described below.

### 2.1. Age at First Malignancy

One of the defining clinical characteristics of cancer predisposition syndromes is the early age at cancer onset compared with the general population. Often leading to cancers occurring in childhood, adolescence, or early adulthood [1,2,3,4].

Because the genetic defect is present in all cells from birth, tumorigenesis may occur earlier than in sporadic cancers, in which multiple somatic mutations must accumulate over time according to the “second hit” framework. Several cancer predisposition syndromes illustrate this early onset pattern. For example, Fanconi anemia, a disorder of DNA repair, is associated with bone marrow failure and an increased risk of AML, typically arising in childhood or adolescence [8].

Cancers in Fanconi anemia occur at much younger ages than in the general population. The peak hazard rate for severe bone marrow failure was 5% per year at age 6.7 years, after which the annual hazard declined substantially. Conversely, the hazard for AML rose after age 10 and then plateaued in the 20- and 30-year-old patients. The hazard for solid tumors increased after age 10 in a more than linear manner, exceeding 10% per year by age 40 [10].

Similarly, Down syndrome predisposes to acute leukemias in early childhood, particularly acute megakaryoblastic leukemia [11]. In contrast, syndromes such as Li–Fraumeni syndrome, caused by germline variants in *TP53*, may present with a broad range of malignancies beginning in childhood but continuing into young adulthood [12,13]. A large international cohort study including more than 3000 individuals carrying germline *TP53* variants demonstrated substantial heterogeneity in age at cancer onset in Li-Fraumeni syndrome. While individuals fulfilling classical clinical criteria more frequently developed early-onset tumors such as sarcomas, brain tumors, and adrenocortical carcinomas, approximately 45% of cancers in carriers with attenuated phenotypes occurred after the age of 45 years, supporting the concept of a broader “Li-Fraumeni spectrum” [14].

Recognition of unusually early cancer onset is therefore an important clinical clue suggesting the presence of an underlying cancer predisposition syndrome.

### 2.2. Cancer Spectrum

Another defining feature of cancer predisposition syndromes is the presence of a characteristic spectrum of malignancies, which often reflects the biological pathway disrupted by the underlying germline mutation. Cancer predisposition syndromes represent a heterogeneous group of disorders that may predispose predominantly to hematologic malignancies, solid tumors, or a combination of both.

Several cancer predisposition syndromes are primarily associated with an increased risk of myeloid neoplasms and bone marrow failure–related malignancies. These include Fanconi anemia, Shwachman–Diamond syndrome, dyskeratosis congenita, and trisomy 21, all of which confer a predisposition to myelodysplastic syndromes (MDS) and acute leukemias, particularly AML or specific leukemia subtypes such as acute megakaryoblastic leukemia in Down syndrome [15,16,17]. In these disorders, the cancer spectrum is closely linked to chronic hematopoietic stress, genomic instability, or impaired stem cell maintenance.

Other cancer predisposition syndromes are characterized by a broader tumor spectrum involving both hematologic and solid malignancies. Constitutional mismatch repair deficiency (CMMRD) is notable for its early onset and wide range of cancers, including high-grade brain tumors, lymphoid malignancies, and gastrointestinal cancers, often occurring as multiple synchronous or metachronous tumors [18]. Similarly, Li–Fraumeni syndrome is associated with a remarkably diverse tumor spectrum encompassing sarcomas, early-onset breast cancer, brain tumors, adrenocortical carcinoma, and leukemias [12,13,14].

In contrast, some cancer predisposition syndromes predispose predominantly to tumors arising in specific tissues or developmental lineages. For example, neurofibromatosis type 1 is characterized by an increased risk of tumors of neural origin, including optic pathway gliomas and malignant peripheral nerve sheath tumors, as well as certain hematologic malignancies such as juvenile myelomonocytic leukemia [19,20].

Several cancer predisposition syndromes exhibit age-dependent shifts in tumor spectrum, with hematologic malignancies often occurring earlier in life and epithelial solid tumors emerging later, as illustrated in Fanconi anemia and telomere biology disorders. Overall, these characteristic patterns of malignancy reflect the molecular pathways altered in each syndrome.

### 2.3. Underlying Biological Mechanisms

At the molecular level, many cancer predisposition syndromes arise from germline alterations in genes that are critical for maintaining genomic stability and regulating cellular proliferation [21]. These include tumor suppressor genes, DNA repair genes, cell-cycle regulators, and pathways involved in telomere maintenance.

Defects in DNA repair pathways represent one of the most prominent mechanisms underlying cancer predisposition syndromes. Fanconi anemia, for example, results from mutations in genes involved in the Fanconi DNA repair pathway, leading to chromosomal instability and hypersensitivity to DNA crosslinking agents [8]. Similarly, ataxia–telangiectasia, caused by pathogenic variants in the ATM gene, disrupts the DNA damage response and predisposes to lymphoid malignancies [22].

Other cancer predisposition syndromes arise from abnormalities in telomere maintenance, as seen in dyskeratosis congenita, a disorder characterized by defective telomere biology and an increased risk of bone marrow failure, MDS, AML, and squamous cell carcinomas [23]. In contrast, syndromes such as Li–Fraumeni syndrome involve germline alterations in key tumor suppressor pathways, particularly TP53, leading to impaired cellular responses to DNA damage and increased susceptibility to multiple malignancies [12,13]. Other cancer predisposition syndromes are inborn errors of immunity, and the increased risk of malignancy is due to defective immunosurveillance. Most malignancies in these conditions are hematological malignancies, especially lymphoma [24,25].

Despite their diversity, many cancer predisposition syndromes share common biological consequences, including genomic instability, impaired DNA damage response, and increased cellular stress, all of which promote malignant transformation.

### 2.4. Absolute and Relative Cancer Risks

Cancer predisposition syndromes are also characterized by substantially increased cancer risks compared with the general population, which may be quantified using cumulative incidence estimates or standardized incidence ratios.

Fanconi anemia is associated with an extremely high risk of hematologic malignancies, with a relative risk estimated at approximately 700-fold for AML and more than 6000-fold for MDS, and a cumulative incidence of AML approaching 15–20% by early adulthood [10]. Similarly, ataxia–telangiectasia carries a markedly increased cancer risk, particularly for lymphoid malignancies, with a standardized incidence ratio of approximately 50–60 for childhood cancers [22].

Disorders of telomere biology, such as dyskeratosis congenita, are associated with a cumulative cancer risk approaching 50% by age 50, primarily involving MDS, AML, and squamous cell carcinomas [23]. Among tumor-predisposition syndromes, Li–Fraumeni syndrome confers one of the highest known cancer risks, with an estimated 50% probability of cancer by age 30 and up to 70–90% over a lifetime [12,13]. In contrast, Down syndrome is associated with a 10–20-fold increased risk of leukemia, particularly acute megakaryoblastic leukemia in childhood [11].

Together, these observations highlight the wide spectrum of cancer susceptibility associated with cancer predisposition syndromes, ranging from moderate increases in risk to extremely high relative risks.

## 3. Polygenic Contribution to Cancer Risk

In addition to cancer predisposition syndromes, which are due to rare, large-effect variants, common variants can also increase the risk of malignancies. Common variants (i.e., single-nucleotide polymorphisms) are defined as having a minor allele frequency >5% and usually have low individual effects. However, as one individual may carry many SNPs associated with an outcome, their cumulative effects can be substantial and are represented by polygenic scores, which sum the effect of all SNPs carried by an individual. Polygenic scores for several malignancies have been developed, including for lung cancer [26], breast cancer [27], prostate cancer, and leukemia [28]. Individuals with a high polygenic score may carry a markedly increased risk of cancer. For example, individuals within the top 10% decile of a polygenic score for lung cancer carry an increased risk of 1.96–2.38 [29]. Despite not being routinely used in clinical practice, these scores could help refine screening approaches, such as the starting age of breast screening [30].

## 4. Clinical Signals of Cancer Predisposition in SCD

### 4.1. Hematological Malignancies and Clonal Hematopoiesis

The strongest signal linking SCD to cancer predisposition concerns hematological malignancies.

Epidemiological studies report an increased incidence of acute leukemias, particularly AML, with standardized incidence ratios ranging from ~3.5 to 10 in large population-based studies [6,31,32,33], and an occurrence at younger ages compared to the general population, with risk increasing from adolescence (around age 15). Early reports, including Schultz et al. [6], first suggested a possible association between SCD and malignancy, which has since been supported by larger cohort studies.

Clinical series further suggest that SCD-associated AML displays distinctive features:

- Frequent association with MDS, with approximately 50% of MDS/AML cases arising from preexisting MDS
- Enrichment in high-risk cytogenetic abnormalities (−7, −5, complex karyotypes, TP53 mutations, 11q23 or KMT2A rearrangements)
- Poor overall prognosis, with median survival reported around 7 months in case series [9]. These observations are consistent with prior reports suggesting that myeloid neoplasms arising in patients with SCD may reflect intrinsic disease-related mechanisms rather than a coincidental association [34].
- In addition, recent studies and reviews have further highlighted the potential contribution of chronic SCD-related complications to leukemogenesis, supporting a multifactorial process involving sustained hematopoietic stress and genomic instability [35,36].

Clonal hematopoiesis has also been identified in SCD patients, sometimes at unexpectedly young ages and involving high-risk mutations such as TP53. These findings support the existence of early genomic instability in the hematopoietic compartment, possibly contributing to leukemogenesis independently of disease-modifying therapies like hydroxyurea or hematopoietic stem cell transplantation.

### 4.2. Solid Tumors

Data on solid tumors in SCD are less consistent.

Current studies report:

• No significant increase or even reduced incidence of solid tumors (32% lower risk in the California cohort) [33]
• Increased risk in selected cohorts, with some studies reporting higher rates of lymphoma and certain solid tumors [32]

Thus, there is currently no clear and consistent solid tumor signature in SCD.

### 4.3. Mechanisms Contributing to Leukemogenesis in SCD

Several biological mechanisms may contribute to the increased risk of malignancy observed in individuals with SCD. Current evidence suggests that chronic inflammation, oxidative stress, hematopoietic stress, premature biological aging, immune dysregulation, and clonal hematopoiesis may collectively create a microenvironment favorable to malignant transformation.

Chronic inflammation results from persistent hemolysis, endothelial activation, and recurrent ischemia–reperfusion injury. Patients with SCD exhibit elevated levels of inflammatory cytokines and activation of several immune and vascular cell types, including neutrophils, monocytes, platelets, and endothelial cells [37,38]. Hemolysis also releases erythrocyte components, such as hemoglobin S, which act as damage-associated molecular patterns and can activate innate immune pathways, including TLR4 signaling [39]. Persistent inflammatory signaling may promote DNA damage and genomic instability, key events in carcinogenesis [40]. In parallel, SCD is characterized by chronic oxidative stress, with increased production of reactive oxygen species (ROS) and elevated markers of lipid peroxidation such as malondialdehyde [41,42]. ROS can induce DNA damage, alter signaling pathways controlling proliferation and apoptosis, and modify the tumor microenvironment, thereby contributing to malignant transformation [43,44].

Another important factor is the intense hematopoietic stress associated with chronic hemolysis. Continuous destruction of red blood cells leads to bone marrow hyperplasia and sustained activation of hematopoietic stem and progenitor cells (HSPCs). Repeated exit of HSPCs from quiescence may increase replication-associated DNA damage [45]. Despite this hyperproliferative state, SCD is also associated with ineffective erythropoiesis, partly due to hemolysis of erythroid precursors in the bone marrow and inflammatory dysregulation of erythropoiesis [46]. Ineffective erythropoiesis and local inflammatory signals may create selective pressures favoring the expansion of hematopoietic clones carrying somatic mutations [47,48].

SCD has also been associated with features of accelerated biological aging. Chronic inflammation, oxidative stress, and sustained hematopoietic turnover may promote premature cellular senescence and exhaustion of the hematopoietic compartment [49]. Several markers of accelerated aging have been described in SCD, including telomere shortening and epigenetic aging signatures [50,51]. Aging of hematopoietic stem cells can impair DNA repair mechanisms and promote clonal selection, thereby increasing susceptibility to malignant transformation [52].

Alterations in immune surveillance may further contribute to cancer risk. Functional asplenia impairs the clearance of abnormal circulating cells and affects both humoral and cellular immune responses [53]. In addition, abnormalities affecting T cells, natural killer cells, and regulatory T cells have been reported [54,55], and recent work suggests that SCD-associated chromatin changes in CD8^+^ T cells may suppress anti-tumor immunity [56].

Finally, clonal hematopoiesis may represent a mechanistic link between chronic hematopoietic stress and leukemogenesis. Clonal hematopoiesis arises from the expansion of hematopoietic clones carrying somatic mutations in genes frequently associated with myeloid malignancies, such as DNMT3A, TET2, ASXL1, and TP53 [57,58]. Several studies support the early occurrence of clonal hematopoiesis in individuals with SCD [59,60,61,62]

## 5. Should Sickle Cell Disease Be Considered a Cancer Predisposition Syndrome?

SCD harbors several features typically associated with cancer predisposition syndromes (Table 1). First, a reproducible epidemiological signal for leukemia has been reported across large population-based studies. Individuals with SCD show a consistently increased risk of acute leukemias, particularly AML, with increased standardized incidence ratios in studies from the United States and the UK [32,33,63].

Second, malignancies appear to occur at an earlier age. Leukemias reported in individuals with SCD frequently arise during adolescence or early adulthood, which is substantially earlier than the typical age of onset observed in the general population [64]. Studies of clonal hematopoiesis also support this earlier age of onset.

Third, clinical series suggest that leukemias arising in the context of SCD may display distinctive biological features. SCD-associated AML often develops from pre-existing myelodysplastic syndromes and is enriched in high-risk cytogenetic or molecular abnormalities, including −7/del(7q), −5/del(5q), complex karyotypes, and mutations involving genes such as *TP53* or *KMT2A*.

Finally, several biological mechanisms plausibly support a role for SCD in leukemogenesis. Although it is not a cancer predisposition syndrome caused by germline mutations in tumor suppressor or DNA repair genes, multiple biological mechanisms disrupted in SCD overlap with those observed in inherited cancer predisposition syndromes. Chronic hemolysis, ongoing inflammation, and recurrent microvascular ischemia in SCD induce hematopoietic stress and increased stem cell turnover, promoting clonal hematopoiesis, which is also a hallmark of bone marrow failure syndromes such as Fanconi anemia, Shwachman–Diamond syndrome, and dyskeratosis congenita. Furthermore, oxidative stress and repeated DNA damage in SCD generate genomic instability, echoing the defects in DNA repair pathways characteristic of Fanconi anemia, CMMRD, and Li–Fraumeni syndrome. Accelerated telomere attrition and premature hematopoietic aging, observed in SCD, parallel the mechanisms driving leukemogenesis in telomere biology disorders. Finally, persistent inflammatory signaling in SCD alters the bone marrow microenvironment and may favor clonal expansion, a feature shared with certain cancer predisposition syndromes, including NF1 and CMMRD. Collectively, these overlapping mechanisms suggest that, although the initiating lesions differ, SCD creates a hematopoietic milieu conducive to leukemogenesis similar to that seen in classical cancer predisposition syndromes [9,59,65].

Emerging data suggest that sickle cell trait may also be associated with a modest and heterogeneous cancer risk, particularly for specific malignancies such as renal cancer, although findings remain inconsistent [66]. This observation supports the hypothesis of a continuum of cancer susceptibility related to hemoglobin S rather than a binary distinction between health and disease.

However, significant differences with cancer predisposition syndromes must be acknowledged. Perhaps most important, the magnitude of cancer risk in SCD appears lower than that of most cancer predisposition syndromes. In contrast to high-penetrance syndromes such as Li-Fraumeni syndrome, in which lifetime cancer risk may exceed 70–90%, the overall absolute cancer risk associated with SCD is substantially lower [20,21]. AML cases remain rare in individuals with SCD. The relative risk of cancer in SCD appears to be closer to that conferred by polygenic predisposition driven by common variations. In addition, epidemiological data remain somewhat inconsistent across studies. Recent systematic reviews also highlight the heterogeneity of cancer risk across hemoglobinopathies, with variable estimates depending on study design and population [67]. While large cohorts from the USA and the UK have demonstrated an increased leukemia risk, other cohorts, such as the Italian multicenter study reported by Origa and colleagues, did not identify leukemia cases in smaller populations. In addition, recent real-world data from a large North Carolina cohort reported measurable prevalence of both hematologic and solid malignancies, but also highlighted substantial variability across populations and study designs [33,63,64,68]. SCD has a high variability in terms of clinical severity. Some individuals have few or no complications, while others may have multiple chronic organ involvements. This heterogeneity is poorly understood. Whether some modifiers of cancer risk exist in SCD remains to be investigated and may allow for refining risk prediction.

Beyond cancer incidence, SCD may also influence cancer outcomes. Several reports suggest poorer survival in patients with SCD-associated malignancies, particularly AML, potentially due to more aggressive disease biology, higher prevalence of adverse cytogenetic features, and challenges in delivering intensive therapies in the context of underlying organ dysfunction [33].

An important distinction between SCD and classical cancer predisposition syndromes lies in the potential impact of therapeutic interventions on cancer risk. In contrast to CPS, where the underlying genetic defect is not modifiable, several treatments used in SCD may alter the hematopoietic environment and thus influence leukemogenesis. On one hand, disease-modifying therapies such as hydroxyurea or curative approaches including hematopoietic stem cell transplantation and gene therapy may reduce chronic inflammation, hemolysis, and hematopoietic stress, potentially mitigating the biological drivers of clonal evolution. On the other hand, these interventions involve cytotoxic exposure or genotoxic conditioning, which may promote the emergence or expansion of pre-existing mutated clones. This dual effect complicates the interpretation of cancer risk in SCD and further distinguishes it from classical CPS. In this context, cases of secondary malignancies following allogeneic transplantation have been reported, and the potential role of conditioning regimens, including low-dose irradiation, in promoting malignant transformation of pre-damaged host cells has been discussed [62,69,70,71,72,73,74]. Overall, these observations support a dynamic and treatment-modulated model of cancer risk in SCD rather than a fixed predisposition state.

Furthermore, no clearly defined cancer spectrum has been established beyond the predominance of AML. Data regarding solid tumors remains heterogeneous and inconclusive, which limits the classification of SCD as a classical cancer predisposition syndrome. Interpretation is also complicated by treatment-related factors. Some leukemias have been reported following hematopoietic stem cell transplantation or gene therapy, making it difficult to distinguish disease-related predisposition from therapy-associated leukemogenesis [6,69,70,71,72,73].

Taken together, these observations suggest that SCD may be best conceptualized as a specific condition associated with increased risk of malignancies—especially AML—rather than a typical cancer predisposition syndrome.

## 6. Conclusions

Current evidence supports an increased risk of AML beginning in childhood, with specific features in individuals with SCD, and several mechanisms may contribute to this risk. These characteristics are shared with many cancer predisposition syndromes. However, substantial differences exist, the main one being the much lower relative risk of cancer in SCD than in most cancer predisposition syndromes. As such, although the absolute risk remains unknown, SCD-associated AML remains a rare event. Although clinicians should be aware of the increased risk of AML in individuals with SCD, this condition appears to have significant features that distinguish it from most cancer predisposition syndromes.

## Figures and Tables

**Table 1:** Comparative features of cancer predisposition syndromes and sickle cell disease.

Feature	Cancer Predisposition Syndromes	Sickle Cell Disease
**Age at first cancer**	Often childhood or early adulthood due to constitutional genetic defects. Age at onset varies by syndrome, ranging from infancy (e.g., Down syndrome-associated leukemia) to adulthood (e.g., telomere biology disorders).	Malignancies reported mainly in adolescence or young adulthood; cancers in early childhood are uncommon.
**Cancer spectrum**	**Predisposition to hematologic malignancies:** • Fanconi anemia: AML • CMMRD: T-NHL, T-ALL, AML, high-grade gliomas and GI cancers • Schwachman–Diamond syndrome: MDS followed by AML • Dyskeratosis congenita: MDS, leukemia • Down syndrome (T21): AML and ALL	Reported cancers are predominantly hematologic, particularly AML and MDS, enriched in high-risk cytogenetic or molecular abnormalities, including −7/del(7q), −5/del(5q), complex karyotypes, and mutations involving genes such as *TP53* or *KMT2A*. Solid tumors have occasionally been described.
	**Predisposition to solid tumors:** • Li–Fraumeni syndrome: breast cancer, sarcoma, osteosarcoma, brain tumor, adrenocortical carcinoma, low hypodiploid B-ALL, MDS/AML • Neurofibromatosis type 1: JMML, rhabdomyosarcoma, malignant peripheral nerve sheath tumor (MPNST), optic pathway glioma • Beckwith–Wiedemann syndrome	
**Underlying biological mechanisms**	Germline alterations affecting key pathways involved in genome maintenance and cellular proliferation: • DNA repair defects: Fanconi anemia • DNA damage response: Ataxia–telangiectasia • Telomere biology disorders: Dyskeratosis congenita • Tumor suppressor pathways: TP53 in Li–Fraumeni syndrome	No germline cancer predisposition gene. Proposed mechanisms include: • chronic inflammation • hematopoietic stress from chronic hemolysis • clonal hematopoiesis • accumulated genomic damage in hematopoietic stem cells
**Relative cancer risk**	**Very-high-risk syndromes:** • Fanconi anemia: AML risk ~700×, MDS risk >6000× • Li–Fraumeni syndrome: lifetime cancer risk 70–90%	Moderately increased risk of malignancy, particularly for myeloid neoplasms
	**High-risk syndromes:** • Ataxia–telangiectasia: overall cancer SIR ~50–60 • Dyskeratosis congenita: ~50% lifetime cancer risk	
	**Moderate-risk syndromes:** • Down syndrome: 10–20× increased leukemia risk	
**Nature of predisposition**	Inherited germline variants in cancer predisposition genes	Secondary cancer susceptibility related to chronic disease biology rather than a classical cancer predisposition syndrome

## Data Availability

No new data were created or analyzed in this study. Data sharing is not applicable to this article.

## Abbreviations

The following abbreviations are used in this manuscript:

SCD	Sickle Cell Disease
AML	Acute Myeloid Leukemia
HbSS	Hemoglobin S
MDS	Myelodysplastic Syndrome
CMMRD	Constitutional Mismatch Repair Deficiency
CH	Clonal Hematopoiesis
ROS	Reactive Oxygen Species
HSPCs	Hematopoietic Stem and Progenitor Cells

## References

[1] Zhang J., Walsh M.F., Wu G., Edmonson M.N., Gruber T.A., Easton J., Hedges D., Ma X., Zhou X., Yergeau D.A. (2015). Germline Mutations in Predisposition Genes in Pediatric Cancer. N. Engl. J. Med..

[2] Gargallo P., Oltra S., Yáñez Y., Juan-Ribelles A., Calabria I., Segura V., Lázaro M., Balaguer J., Tormo T., Dolz S. (2021). Germline Predisposition to Pediatric Cancer, from Next Generation Sequencing to Medical Care. Cancers.

[3] Byrjalsen A., Hansen T.V.O., Stoltze U.K., Mehrjouy M.M., Barnkob N.M., Hjalgrim L.L., Mathiasen R., Lautrup C.K., Gregersen P.A., Hasle H. (2020). Nationwide germline whole genome sequencing of 198 consecutive pediatric cancer patients reveals a high incidence of cancer prone syndromes. PLoS Genet..

[4] Mody R.J., Wu Y.-M., Lonigro R.J., Cao X., Roychowdhury S., Vats P., Frank K.M., Prensner J.R., Asangani I., Palanisamy N. (2015). Integrative Clinical Sequencing in the Management of Refractory or Relapsed Cancer in Youth. JAMA.

[5] Garutti M., Foffano L., Mazzeo R., Michelotti A., Da Ros L., Viel A., Miolo G., Zambelli A., Puglisi F. (2023). Hereditary Cancer Syndromes: A Comprehensive Review with a Visual Tool. Genes.

[6] Schultz W.H., Ware R.E. (2003). Malignancy in patients with sickle cell disease. Am. J. Hematol..

[7] Kratz C.P. (2025). Re-envisioning genetic predisposition to childhood and adolescent cancers. Nat. Rev. Cancer.

[8] Alter B.P., Giri N., Savage S.A., Peters J.A., Loud J.T., Leathwood L., Carr A.G., Greene M.H., Rosenberg P.S. (2010). Malignancies and survival patterns in the National Cancer Institute inherited bone marrow failure syndromes cohort study. Br. J. Haematol..

[9] Casadessus E., Saby M., Forté S., Pastore Y., Lavallée V.-P., Pincez T. (2025). When Blood Disorders Meet Cancer: Uncovering the Oncogenic Landscape of Sickle Cell Disease. J. Clin. Med..

[10] Alter B.P. (2014). Fanconi anemia and the development of leukemia. Best Pract. Res. Clin. Haematol..

[11] Roberts I., Izraeli S. (2014). Haematopoietic development and leukaemia in Down syndrome. Br. J. Haematol..

[12] Bougeard G., Renaux-Petel M., Flaman J.-M., Charbonnier C., Fermey P., Belotti M., Gauthier-Villars M., Stoppa-Lyonnet D., Consolino E., Brugières L. (2015). Revisiting Li-Fraumeni Syndrome From TP53 Mutation Carriers. J. Clin. Oncol..

[13] Malkin D. (2011). Li-fraumeni syndrome. Genes Cancer.

[14] Kratz C.P., Freycon C., Maxwell K.N., Nichols K.E., Schiffman J.D., Evans D.G., Achatz M.I., Savage S.A., Weitzel J.N., Garber J.E. (2021). Analysis of the Li-Fraumeni Spectrum Based on an International Germline TP53 Variant Data Set: An International Agency for Research on Cancer TP53 Database Analysis. JAMA Oncol..

[15] Ripperger T., Bielack S.S., Borkhardt A., Brecht I.B., Burkhardt B., Calaminus G., Debatin K.-M., Deubzer H., Dirksen U., Eckert C. (2017). Childhood cancer predisposition syndromes-A concise review and recommendations by the Cancer Predisposition Working Group of the Society for Pediatric Oncology and Hematology. Am. J. Med. Genet. A.

[16] Nakano Y., Rabinowicz R., Malkin D. (2023). Genetic predisposition to cancers in children and adolescents. Curr. Opin. Pediatr..

[17] Coury S.A., Schneider K.A., Schienda J., Tan W.-H. (2018). Recognizing and Managing Children with a Pediatric Cancer Predisposition Syndrome: A Guide for the Pediatrician. Pediatr. Ann..

[18] Ripperger T., Schlegelberger B. (2016). Acute lymphoblastic leukemia and lymphoma in the context of constitutional mismatch repair deficiency syndrome. Eur. J. Med. Genet..

[19] Niemeyer C.M. (2018). JMML genomics and decisions. Hematol. Am. Soc. Hematol. Educ. Program.

[20] Evans D.G.R., Salvador H., Chang V.Y., Erez A., Voss S.D., Schneider K.W., Scott H.S., Plon S.E., Tabori U. (2017). Cancer and Central Nervous System Tumor Surveillance in Pediatric Neurofibromatosis 1. Clin. Cancer Res..

[21] Rahman N. (2014). Realizing the promise of cancer predisposition genes. Nature.

[22] Dutzmann C.M., Spix C., Popp I., Kaiser M., Erdmann F., Erlacher M., Dörk T., Schindler D., Kalb R., Kratz C.P. (2022). Cancer in Children With Fanconi Anemia and Ataxia-Telangiectasia-A Nationwide Register-Based Cohort Study in Germany. J. Clin. Oncol..

[23] Savage S.A. (2022). Dyskeratosis congenita and telomere biology disorders. Hematol. Am. Soc. Hematol. Educ. Program.

[24] Mortaz E., Tabarsi P., Mansouri D., Khosravi A., Garssen J., Velayati A., Adcock I.M. (2016). Cancers Related to Immunodeficiencies: Update and Perspectives. Front. Immunol..

[25] Pai S.-Y., Lurain K., Yarchoan R. (2021). How immunodeficiency can lead to malignancy. Hematol. Am. Soc. Hematol. Educ. Program.

[26] Galal B., Dennis J., Antoniou A.C., Harrison H. (2026). The current state of polygenic scores for the development of lung cancer: A systematic review and validation in UK Biobank. Br. J. Cancer.

[27] Li J.L., Zhang H., Wang X., Jia G., McClellan J.C., Guo W., Sun Y., Fiorica P.N., Ambs S., Barnard M.E. (2026). Improved polygenic risk prediction models for breast cancer subtypes in women of African ancestry. Nat. Genet..

[28] Jeon S., Lo Y.C., Morimoto L.M., Metayer C., Ma X., Wiemels J.L., de Smith A.J., Chiang C.W.K. (2023). Evaluating genomic polygenic risk scores for childhood acute lymphoblastic leukemia in Latinos. HGG Adv..

[29] Dai J., Lv J., Zhu M., Wang Y., Qin N., Ma H., He Y.-Q., Zhang R., Tan W., Fan J. (2019). Identification of risk loci and a polygenic risk score for lung cancer: A large-scale prospective cohort study in Chinese populations. Lancet Respir. Med..

[30] Mavaddat N., Michailidou K., Dennis J., Lush M., Fachal L., Lee A., Tyrer J.P., Chen T.-H., Wang Q., Bolla M.K. (2019). Polygenic Risk Scores for Prediction of Breast Cancer and Breast Cancer Subtypes. Am. J. Hum. Genet..

[31] Dawkins F.W., Kim K.S., Squires R.S., Chisholm R., Kark J.A., Perlin E., Castro O. (1997). Cancer incidence rate and mortality rate in sickle cell disease patients at Howard University Hospital: 1986–1995. Am. J. Hematol..

[32] Seminog O.O., Ogunlaja O.I., Yeates D., Goldacre M.J. (2016). Risk of individual malignant neoplasms in patients with sickle cell disease: English national record linkage study. J. R. Soc. Med..

[33] Brunson A., Keegan T.H.M., Bang H., Mahajan A., Paulukonis S., Wun T. (2017). Increased risk of leukemia among sickle cell disease patients in California. Blood.

[34] Li Y., Maule J., Neff J.L., McCall C.M., Rapisardo S., Lagoo A.S., Yang L.-H., Crawford R.D., Zhao Y., Wang E. (2019). Myeloid neoplasms in the setting of sickle cell disease: An intrinsic association with the underlying condition rather than a coincidence; report of 4 cases and review of the literature. Mod. Pathol..

[35] Cannas G., Poutrel S., Heiblig M., Labussière H., Larcher M.-V., Thomas X., Hot A. (2023). Sickle cell disease and acute leukemia: One case report and an extensive review. Ann. Hematol..

[36] Cannas G., Elhamri M., Thomas X. (2024). Is There any Relationship Between the Repeated Complications of Sickle Cell Disease and the Potential Development of Acute Leukemia?. Oncol. Ther..

[37] Conran N., Belcher J.D. (2018). Inflammation in sickle cell disease. Clin. Hemorheol. Microcirc..

[38] Zhang D., Xu C., Manwani D., Frenette P.S. (2016). Neutrophils, platelets, and inflammatory pathways at the nexus of sickle cell disease pathophysiology. Blood.

[39] Allali S., Rignault-Bricard R., de Montalembert M., Taylor M., Bouceba T., Hermine O., Maciel T.T. (2022). HbS promotes TLR4-mediated monocyte activation and proinflammatory cytokine production in sickle cell disease. Blood.

[40] Kay J., Thadhani E., Samson L., Engelward B. (2019). Inflammation-induced DNA damage, mutations and cancer. DNA Repair.

[41] Nur E., Biemond B.J., Otten H.-M., Brandjes D.P., Schnog J.-J.B., CURAMA Study Group (2011). Oxidative stress in sickle cell disease; pathophysiology and potential implications for disease management. Am. J. Hematol..

[42] Antwi-Boasiako C., Dankwah G.B., Aryee R., Hayfron-Benjamin C., Donkor E.S., Campbell A.D. (2019). Oxidative Profile of Patients with Sickle Cell Disease. Med. Sci..

[43] Iqbal M.J., Kabeer A., Abbas Z., Siddiqui H.A., Calina D., Sharifi-Rad J., Cho W.C. (2024). Interplay of oxidative stress, cellular communication and signaling pathways in cancer. Cell Commun. Signal..

[44] Liang X., Weng J., You Z., Wang Y., Wen J., Xia Z., Huang S., Luo P., Cheng Q. (2025). Oxidative stress in cancer: From tumor and microenvironment remodeling to therapeutic frontiers. Mol. Cancer.

[45] Walter D., Lier A., Geiselhart A., Thalheimer F.B., Huntscha S., Sobotta M.C., Moehrle B., Brocks D., Bayindir I., Kaschutnig P. (2015). Exit from dormancy provokes DNA-damage-induced attrition in haematopoietic stem cells. Nature.

[46] El Hoss S., Shangaris P., Brewin J., Psychogyiou M.E., Ng C., Pedler L., Rooks H., Gotardo É.M.F., Gushiken L.F.S., Brito P.L. (2025). Reduced GATA1 levels are associated with ineffective erythropoiesis in sickle cell anemia. Haematologica.

[47] Attardi E., Corey S.J., Wlodarski M.W. (2024). Clonal hematopoiesis in children with predisposing conditions. Semin. Hematol..

[48] Cai Z., Kotzin J.J., Ramdas B., Chen S., Nelanuthala S., Palam L.R., Pandey R., Mali R.S., Liu Y., Kelley M.R. (2018). Inhibition of Inflammatory Signaling in Tet2 Mutant Preleukemic Cells Mitigates Stress-Induced Abnormalities and Clonal Hematopoiesis. Cell Stem Cell.

[49] Li X., Wang J., Hu L., Cheng T. (2025). How age affects human hematopoietic stem and progenitor cells and the strategies to mitigate aging. Exp. Hematol..

[50] Mekontso Dessap A., Cecchini J., Chaar V., Marcos E., Habibi A., Bartolucci P., Ghaleh B., Galacteros F., Adnot S. (2017). Telomere attrition in sickle cell anemia. Am. J. Hematol..

[51] Lê B.M., Hatch D., Yang Q., Shah N., Luyster F.S., Garrett M.E., Tanabe P., Ashley-Koch A.E., Knisely M.R. (2024). Characterizing epigenetic aging in an adult sickle cell disease cohort. Blood Adv..

[52] Colom Díaz P.A., Mistry J.J., Trowbridge J.J. (2023). Hematopoietic stem cell aging and leukemia transformation. Blood.

[53] Brousse V., Buffet P., Rees D. (2014). The spleen and sickle cell disease: The sick(led) spleen. Br. J. Haematol..

[54] Patel S., Chandrakar D., Wasnik P.N., Nayak S., Shah S., Nanda R., Mohapatra E. (2023). Altered T-cell profile in sickle cell disease. Biomark. Med..

[55] Vingert B., Tamagne M., Desmarets M., Pakdaman S., Elayeb R., Habibi A., Bernaudin F., Galacteros F., Bierling P., Noizat-Pirenne F. (2014). Partial dysfunction of Treg activation in sickle cell disease. Am. J. Hematol..

[56] Zhao Z., Hu B., Deng Y., Soeung M., Yao J., Bei L., Zhang Y., Gong P., Huang L.A., Jiang Z. (2025). Sickle cell disease induces chromatin introversion and ferroptosis in CD8+ T cells to suppress anti-tumor immunity. Immunity.

[57] Jaiswal S., Ebert B.L. (2019). Clonal hematopoiesis in human aging and disease. Science.

[58] Gibson C.J., Steensma D.P. (2018). New Insights from Studies of Clonal Hematopoiesis. Clin. Cancer Res..

[59] Pincez T., Lee S.S.K., Ilboudo Y., Preuss M., d’Alexandry d’Orengiani A.-L.P.H., Bartolucci P., Galactéros F., Joly P., Bauer D.E., Loos R.J.F. (2021). Clonal hematopoiesis in sickle cell disease. Blood.

[60] Ulloa J., Wuichet K., Rashkin S.R., Pershad Y., Shore C., Vlasschaert C., Rodeghier M., Yao Y., Gordeuk V.R., Shah B.N. (2026). Increased prevalence of clonal hematopoiesis in children with sickle cell disease. Blood.

[61] Weeks L., Fitzhugh C., Pollock S., Osei M., Rickles-Young M., Murdock H.M., Townsend M., Reilly C., Dinardo C., Sabino E. (2025). Sickle cell disease is associated with early-onset clonal hematopoiesis involving DNA damage response pathway mutations. Blood.

[62] Spencer Chapman M., Cull A.H., Ciuculescu M.F., Esrick E.B., Mitchell E., Jung H., O’Neill L., Roberts K., Fabre M.A., Williams N. (2023). Clonal selection of hematopoietic stem cells after gene therapy for sickle cell disease. Nat. Med..

[63] Easaw S., Kayle M., Fisher E., Reyes C., Crego N., Strouse J.J., Copelan E.A., Desai P.C. (2024). Prevalence of Malignancies in Patients with Sickle Cell Disease in North Carolina. Blood.

[64] Origa R., Gianesin B., Longo F., Di Maggio R., Cassinerio E., Gamberini M.R., Pinto V.M., Quarta A., Casale M., La Nasa G. (2023). Incidence of cancer and related deaths in hemoglobinopathies: A follow-up of 4631 patients between 1970 and 2021. Cancer.

[65] Ribeil J.-A. (2022). Primary myelofibrosis in untreated sickle cell disease: Are adult patients at higher risk for developing hematological myeloid neoplasms?. Am. J. Hematol..

[66] Richards S., Elkomi R., Tabraiz S.A., Kerolle E., Zinabu S., Ali A., Michael M. (2025). Sickle cell trait and the risk for malignancy. J. Natl. Med. Assoc..

[67] Merolle L., Ghirotto L., Schiroli D., Balestra G.L., Venturelli F., Bassi M.C., Di Bartolomeo E., Baricchi R., Marraccini C. (2025). Risk of cancer in patients with thalassemia and sickle cell disease: A systematic review. Ann. Med..

[68] Mercier-Ross J., Yang Z., Zebrowski A., Curtis S., Hua M., Glassberg J. (2026). Incidence of Hematologic and Solid Tumors in Adults With Sickle Cell Disease. Am. J. Hematol..

[69] Goyal S., Tisdale J., Schmidt M., Kanter J., Jaroscak J., Whitney D., Bitter H., Gregory P.D., Parsons G., Foos M. (2022). Acute Myeloid Leukemia Case after Gene Therapy for Sickle Cell Disease. N. Engl. J. Med..

[70] Hsieh M.M., Bonner M., Pierciey F.J., Uchida N., Rottman J., Demopoulos L., Schmidt M., Kanter J., Walters M.C., Thompson A.A. (2020). Myelodysplastic syndrome unrelated to lentiviral vector in a patient treated with gene therapy for sickle cell disease. Blood Adv..

[71] Ghannam J.Y., Xu X., Maric I., Dillon L., Li Y., Hsieh M.M., Hourigan C.S., Fitzhugh C.D. (2020). Baseline TP53 mutations in adults with SCD developing myeloid malignancy following hematopoietic cell transplantation. Blood.

[72] Eapen M., Brazauskas R., Walters M.C., Bernaudin F., Bo-Subait K., Fitzhugh C.D., Hankins J.S., Kanter J., Meerpohl J.J., Bolaños-Meade J. (2019). Effect of donor type and conditioning regimen intensity on allogeneic transplantation outcomes in patients with sickle cell disease: A retrospective multicentre, cohort study. Lancet Haematol..

[73] Janakiram M., Verma A., Wang Y., Budhathoki A., Suarez Londono J., Murakhovskaya I., Braunschweig I., Minniti C.P. (2018). Accelerated leukemic transformation after haplo-identical transplantation for hydroxyurea-treated sickle cell disease. Leuk. Lymphoma.

[74] Eapen M., Brazauskas R., Williams D.A., Walters M.C., St Martin A., Jacobs B.L., Antin J.H., Bona K., Chaudhury S., Coleman-Cowger V.H. (2023). Secondary Neoplasms After Hematopoietic Cell Transplant for Sickle Cell Disease. J. Clin. Oncol..

